# Note from the Editor: Science Education and Outreach Web Page

**Published:** 2011-01

We at *EHP* are pleased to announce the launch of our enhanced Science Education and Outreach web page. We have designed the new web page to make it easier to find and use our resources through features including:

- Theme-based listing of lessons under the categories of air, water, land, food, climate change, and environmentally related diseases
- Related *EHP* articles for use as supplementary reading materials
- An interactive format that allows users to post comments concerning individual lessons
- Science fair project ideas based on selected individual lessons
- Announcements of *EHP-*sponsored professional development workshops for teachers and internships for high-school students.

Our Science Education Program was initiated in 2005 with the objective of providing classroom resources for middle- and high-school science teachers and undergraduate faculty. More than 100 lessons based on research and news published in *EHP* were developed to help teach scientific concepts in biology, physics, chemistry, toxicology, and environmental science, with environmental health as the overarching theme. With our newly expanded Science Education Program we are updating existing lessons, publishing new lessons on current and emerging topics, and translating selected lessons into Spanish.

Starting in 2009, we also launched an Outreach Program to promote professional development, through which we provide materials for and sponsor workshops related to the preparation of research papers and scientific writing. We will be publishing more information about these workshops on our new Science Education and Outreach web page.

We are excited by the opportunity to work closely with students, teachers, and others to advance science education nationally and internationally. We hope our new web page will be a vibrant forum for discussion and sharing of experiences related to science education in the classroom. Please contact Banalata Sen (senb@niehs.nih.gov) with any comments and suggestions concerning the new page.

